# Supplementary material for: Complexome profiling on the Chlamydomonas lpa2 mutant reveals insights into PSII biogenesis and new PSII associated proteins
Source: J Exp Bot. 2021 Aug 26;73(1):245–62. doi: 10.1093/jxb/erab390 (PMC8730698; doi:10.1093/jxb/erab390)
Supplement: erab390_suppl_Supplementary_Dataset_S1 [file erab390_suppl_supplementary_dataset_s1.zip › Supplemental Dataset 1 - Excel List and all profiles/plots/RPL7A_Cre12.g529651.html]

Trivial name: RPL7A  
  
Euclidean distance: 6521.76  
Mean Intensity (WT): 831.26  
Mean Intensity (Mut): 541.42  
Distance: 7.85  
  
MapMan: protein.synthesis.ribosomal protein.eukaryotic.60S subunit.L7A  
  
p value of intensity sums Welch test: 0.6223
